# Management for Caries Prevention in ADHD Children

**DOI:** 10.3390/ijerph19127455

**Published:** 2022-06-17

**Authors:** Elzbieta Paszynska, Anna Krahel, Malgorzata Pawinska, Monika Dmitrzak-Węglarz, Aleksandra Perczak, Agnieszka Słopień, Maria Gawriolek

**Affiliations:** 1 Department of Integrated Dentistry, Poznan University of Medical Sciences, 60-812 Poznan, Poland; akrahel@wp.pl (A.K.); mgawriolek@gmail.com (M.G.); 2 Department of Integrated Dentistry, Medical University in Bialystok, 15-276 Bialystok, Poland; malgosiapaw@interia.pl; 3 Psychiatric Genetics Unit, Department of Psychiatry, Poznan University of Medical Sciences, 60-806 Poznan, Poland; mweglarz@ump.edu.pl; 4 Dental Practice Aleksadra Perczak, Nowa Street 15, 85-119 Bydgoszcz, Poland; euro-dent@wp.pl; 5 Department of Child and Adolescent Psychiatry, Poznan University of Medical Sciences, 60-572 Poznan, Poland; asrs@wp.pl

## 1. Introduction

Emotional Dysregulations (ED) represent a major health risk present in about 5% of children and are associated with diverse forms of childhood psychiatric disorders and symptoms such as Attention-Deficit/Hyperactivity Disorder (ADHD) [1]. Early Childhood Caries (ECC) is still one of the most prevalent disease that can lead to serious health issues in young growing children worldwide [2]. It is possible that the association between ADHD and ECC is more pronounced in children with clinically relevant emotional deficits as in the case of ADHD but may be unnoticeable during typical dental care. Studies relating to oral cavity of children with ADHD are few and mostly of limited assessment.

### 1.1. The Incidence of Carious Lesions among ADHD Children

Within the published studies, children over 10 years old prevail, which makes it difficult to assess the oral health only on the basis of permanent teeth. Studies regarding younger children, i.e., those under 8 years of age with primary dentition, lack definitiveness. This is most probably associated with the point of age at which the diagnosis is made, i.e., between the ages of 6 and 8.

Literature regarding children affected by ADHD suggests that caries prevalence in increased in this group [3,4,5,6]. Broadbent et al. [3] showed that children with ADHD are twice as likely to be in the high-caries risk group. The values of DMFT were over 2.0 [7,8]. A study conducted on a group of Swedish children with ADHD showed that the mean value of DMFT was 2.8 ± 4, whereas its value was within the range of 2.2 ± 3.2 for healthy children [4]. Paszynska et al. [6] showed that DMFT values were 0.8 ± 0.9, whereas dmft values were 4.4 ± 4. However, it should be underlined that the study included younger children, and the mean value for age was 8. In previous studies regarding ADHD, the age of children was higher [7,8,9]. The mean value for age of the studied children was higher than 10 (approximately >12 years old.), which makes it impossible to compare the state of deciduous teeth, and caries was assessed in permanent teeth only. In addition, small sample size (21–50 children) constituted a weak point of the studies [4,5]. It can also be noted that the number of teeth with new carious cavities decreases with age, even though no differences in oral hygiene and diet are observed between healthy children and those affected by ADHD [5]. A partial remission of typical ADHD symptoms in adolescence may exert a positive influence on an improvement in oral hygiene and diet normalization in adolescents. The results of the most relevant clinical research are included in Table 1.

### 1.2. Evaluation of Caries Prevention among ADHD Children

Authors of these and others studies underline that caries development is influenced by a significantly more frequent intake of cariogenic carbohydrates, irregularity in eating patterns as well as less systematic and shorter brushing [11,12,14]. In the evaluation of oral hygiene, worse parameters for plaque indices have been shown [5,6,7,8,9,12,14]. It seems the view that lack of attention, impulsivity and impaired execution function will interfere with oral hygiene maintenance is correct. Moreover, it seems probable that impairment in emotional processing influences the compulsive intake of cariogenic snacks, and lower ability to self-regulate motivation and impaired temporal behavior organization make it difficult for the patient to predict consequences of their actions, which is crucial for maintaining oral health.

Increased plaque accumulation and gingival inflammation may result from the high-carbohydrate diet that is preferred by such patients. There is a tendency to eat more frequently and snack between meals in children with ADHD. If there are no limits placed by the caretakers and if they do not control the process, the increase in the number of meals may exert a huge influence on the progress of caries in deciduous and permanent dentition. However, there are conflicting views in the literature—on the one hand, some researchers confirm that irregular brushing influences high caries prevalence (irrespective of background, education level, and financial status); on the other, other researchers do not observe such correlation [4]. Bretz et al. (2018) discussed the view that a high-carbohydrate diet promotes the development of Streptococcus sobrinus, which are better suited for plaque colonization due to the presence of sucrose in the oral cavity. These authors made the assumption that other acid-forming microorganisms are less active if Streptococcus sobrinus thrives first. However, if the availability of sugars in the oral cavity is changed, the proportion of cariogenic species return to normal. The influence of sugar on caries risk is classified as a moderated caries risk factor for the majority of populations who are under constant exposure to remineralizing agents. It should also be taken into account that people affected by ADHD have larger problems with solving complicated problems and detecting the cause–effect relationship. Deficits regarding the execution function make it more difficult for the child to perform activities that involved planning and organization if they are not motivated and stimulated properly.

A higher number of parent–child conflicts has been reported in families with a child affected by ADHD, which may also influence oral health behaviors. Quite significant reduction in child functioning in comparison to a healthy child, requires the parents to support the child in a variety of everyday chores, including oral hygiene maintenance and keeping the dietary regime. It should be an alarming sign for those responsible for planning oral health and should motivate them to keep track of dental appointments in such children. According to questionnaire-based studies, the numbers of appointments differ between healthy children and ADHD-affected children [8,12]. The results of questionnaires show that families with ADHD require additional support and should be kept under control and the breaks between dental appointments should be shorter in order to prevent the development of caries, as these groups sow worse oral health behavior patterns. Dental appointments are frequently not planned to control and prevent oral diseases, but they take place due to traumatic incidents and random situations [15,16]. In conclusion, when it comes to oral health in children with ADHD, it can be said that the risk of caries is a multifactorial issue resulting from its etiology, nevertheless, the diagnosis of ADHD may constitute an important (but not the only) criterion for caries risk development.

ADHD may constitute a risk factor for higher caries incidence; however, the role of medications used in such children should not be overlooked [17]. The influence of drugs on stimulated salivary flow is well-known and documented [15]. Stimulant-based drugs such as methylphenidate, dexmethylphenidate, mix amphetamine salts and dextroamphetamine cause a decrease in salivary flow, which in turn may cause oral dryness. Refined carbohydrates and dry mouth constitute strong risk factors for dental caries and periodontal disease. Even though the study by Friedlander and Friedlander [18] did not show any relationship between the drugs and caries, Hidas et al. [12] showed significant differences in salivary flow, oral hygiene indices and caries. The amount of salivary flow was not low enough to show hyposalivation but was significantly lower than in the control group. Such observations regarding lower activity of salivary glands were confirmed by Vafaei et al. [17]. Children with ADHD treated with methylphenidate-based drugs showed lower values of non-stimulated saliva, higher dental caries and plaque indices in comparison to children treated only using nonpharmacological methods [17]. A combination of pharmacotherapy with behavioral and cognitive interventions may prevent common adverse effects and induce better outcomes. Neurofeedback is a non-invasive example method that is based on EEG-reading and is based on learning that depends on instrumental conditioning, in which the desired behaviors are rewarded. Stimulation of desirable brain waves and the inhibition of undesirable brain waves is stimulated. Neurofeedback has been used for a variety of purposes in neurorehabilitation [19].

Additionally, anthropometric studies indicated that children with ADHD may represent a population at risk of overweight [6,20,21]. The candidate factors that might be involved in obesity etiology are still searched for and, in particular, children affected by ADHD might be related to binge eating disorders (BED), impulsiveness and abnormal food behaviors, mainly in the form of periodical episodes [22,23]. It is rather unknown how the abovementioned executive functions could be pronounced in oral health. In the situation of consuming large amount of food, accompanied by loss of control and lack of oral hygiene, they add to demineralization of dental tissue and to gingival inflammation.

To help accelerate progress related to the extension of the diagnostic framework for ADHD patients, there is a need to monitor children at risk of oral diseases. From the patient’s side, the action of arresting dental plaque deposit may start from regular tooth brushing and mouth rinsing [24]. Preventive strategies include toothbrushing with a suitable toothbrush and toothpaste, the use of mouthwashes and oral gels, as well as interdental tooth cleaning using, for example, dental floss or interdental brushes. To improve the convenience and compliance, oral care products have to be selected. Those products can not only stop demineralization and stimulate remineralization but can prevent any side effects in case of swallowing. Biomimetic ingredients should be favored, especially those with proven benefits for oral health and comparable to fluoride in terms of their ability to remineralization but without the risk connected to fluoride [22]. In clinical and experimental research, biomimetic hydroxyapatite and calcium-phosphate oral care products are considered, especially for children and adolescents from high-risk groups [6,25,26,27,28]. A mode of action is known due to the deposition on tooth surfaces of hydroxyapatite particles that form mineral–mineral bridges with enamel crystals, as well as indirectly through the release of calcium and phosphate ions and hydroxyapatite’s buffering properties in acidic environments, i.e., in dental plaque [2,28].

**Figure 1:**
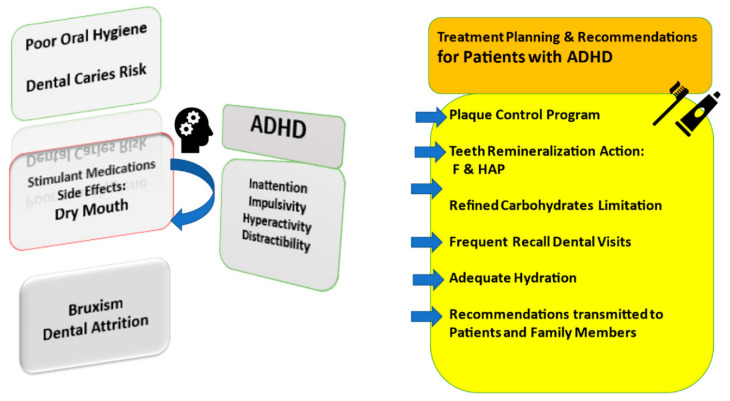
Oral care planning for young patients with ADHD. F—fluoride remineralizing products; HAP—hydroxyapatite oral care remineralizing products.

Professional dental diagnosis desires an accurate severity evaluation. The following methods to detect carious lesions are considered as macroscopic visual examination, bitewing X-ray pictures, tooth separation by orthodontic rubber wedges and non-invasive transillumination of dental tissues. Clinical and/or digital diagnosis methods differ by repeatability and sensitivity; therefore, the combination of two procedures has to be used at the same time. In general, carious lesion activity is the next step, and its assessment will select a proper management strategy such as non-operative care (NOC) or tooth-preserving operative care (TPOC). Only in low caries risk will a dental professional decide on lesion monitoring at recall appointments. In moderate/high caries risks, lesion arresting by remineralization is needed. Remineralization efficacy is possible only when acid and sucrose (refined carbohydrate) intake limitations, oral hygiene (e.g., mouth rinses) and frequency of dental visit improvements occur. The most common dental treatment planning and recommendations are shown in Figure 1.

## 2. Conclusions

The outcomes of the present survey allowed us to draw the following conclusions:

- Clinical data of ADHD trials suggest that effective prevention may depend on level of caries risk, patient’s cooperation and therapeutic management;
- Early strategies for individual patient cases and choices of remineralization products are needed to defend the oral health of children with ADHD against dental caries;
- All abovementioned preventive directions may be applied, and longer follow-up periods are suggested to evaluate the clinical approach in oral health among ADHD patients.

## Figures and Tables

**Table 1:** A synthesis of data obtained from the electronic research organized in PubMed database and Web of Science. The following MeSH and non-MeSH search terms were used: (“ADHD” [MeSH terms], [All fields] OR “Oral Health” [All fields]). “Caries” and “Children and Adolescents” include the following terms: Attention Deficit Disorder with Hyperactivity, Child, Cross-Sectional Studies, Caries Index, Dental Caries Complications, Prevalence. The search selected publications only with an ADHD group of subjects published in English language [].

Authors of Clinical Studies Country	Age Range Min-Max or Mean [years]	Number of ADHD Subjects	Control Group +Yes −No	Oral Examination Methodology	Significant Results for ADHD Group
					Significant Conclusions
Broadbent et al., 2004 [3] New Zeland	11–14	64	+	analysis of dental service records	higher caries experience (odds of 12 times)
					ADHD condition may affect children’s dental caries experience
Bimstein et al., 2008 [10] USA	7.4	25	+	analysis of dental service records	higher prevalence of toothache, bruxism, bleeding gums and oral trauma histories recorded; no differences in plaque accumulation, gingival inflammation, calculus, oral hygiene level, dental caries treatment
					ADHD condition may affect children’s oral health
Blomqvist et al., 2006 [4] Sweden	11	25	+	clinical dental examination, bitewing radiographs, parents’ questionnaire interview	higher caries prevalence, not significant degree of dental anxiety, but differences in behavioral management
					ADHD children desire an intensive oral health control
Blomqvist et al., 2007 [7] Sweden	13	21	+	clinical dental examination, parents’ questionnaire interview	no significant caries experience, poorer oral health behaviors
					ADHD condition indicates for shorter intervals between dental examinations
Blomqvist et al., 2011 [5] Sweden	17	32	+	clinical and radiographic dental examinations	higher caries prevalence and gingival inflammation
					ADHD adolescents desire an intensive oral health control
Chandra et al., 2009 [11] India	8.9	40	+	clinical dental examinations, parents’ questionnaire interview	significant caries in primary dentition, poorer oral hygiene and sweetened consumption control
					ADHD children desire an intensive oral health control
Hidas et al., 2011, 2013 [12,13] Israel	ADHD non-medicated 10.3 ADHD medicated 11.8	31 non-medicated 30 medicated ADHD patients	+	clinical dental examination, plaque index, oral mucosa pH and unstimulated whole salivary flow (USF), parents’ questionnaire interview	in both ADHD groups, no differences in caries incidence, diet/hygiene habits, significant lower USF and higher dental plaque
					ADHD condition may be a factor contributing to caries in older age
Chau et al., 2016 [14] Honk Kong China	12–18	31	+	intraoral dental/periodontal, salivary function, tooth wear examination, parents’ questionnaire interview	no significant differences between children, with or without ADHD, in dental caries, trauma prevalence, periodontal disease, plaque, tooth wear or USF significant difference in gingival bleeding, oral hygiene habits, higher attendance at dental clinic
					poorer oral hygiene, more adverse oral-health attitudes
Begnini et al., 2019 [8] Italy	7–14	51	+	intraoral dental/gingival examination, parents’ questionnaire interview	no differences in dental caries, although visible plaque, gingival bleeding were detected
					ADHD children need supervision on oral health
Ehlers et al., 2019 [9] Germany	9–15	34	+	intraoral dental/gingival, parents questionnaire interview	no differences in oral health; however, higher indices in secondary dentition
					parents/guardians need instructions for better supervision of oral hygiene and dietary habits
Paszynska et al., 2020 [6] Poland	8.2	39	+	physical measurements, clinical dental examination, parents’ questionnaire interview	significant prevalence of abnormal body weight, hip circumference, BMI, caries differences for primary/permanent teeth, primary tooth decay was corelated to sweet consumption
					limiting sugar consumption might be one of preventive point against dental caries and overweight/obesity

Description of the abbreviations: ADHD (Attention-Deficit Disorder with Hyperactivity), USF (unstimulated salivary flow), BMI (Body Mass Index).

## Abbreviations

ADHD—Attention-Deficit Hyperactivity Disorder; BMI—Body Mass Index; BED—binge eating disorders; DMFT/dmft—decay, missing, filled of secondary/primary teeth; DSM-V—Diagnostic and statistical manual of mental disorders (5th ed.); ECC—Early Childhood Caries; ED—Emotional Dysregulations; NOC—non-operative care; TPOC—tooth preserving operative care; USF—unstimulated salivary flow; WHO—World Health Organization.
